# Supplementary material for: Mechanisms governing the pioneering and redistribution capabilities of the non-classical pioneer PU.1
Source: Nat Commun. 2020 Jan 21;11:402. doi: 10.1038/s41467-019-13960-2 (PMC6972792; doi:10.1038/s41467-019-13960-2)
Supplement: Supplementary file 7 — Source data [file 41467_2019_13960_MOESM7_ESM.zip › Source_Data/FigureS2/FigureS2B_MotifScanOutput/KmeansCluster7/homerResults/motif20.similar.html]

motif20

## Information for motif20

A
G
T
C
G
C
T
A
A
G
T
C
T
G
C
A
G
A
T
C
G
A
C
T
A
G
C
T
A
G
C
T
A
G
C
T
T
G
C
A
C
T
G
A
T
G
C
A
T
A
G
C
  
Reverse Opposite:  

A
T
C
G
A
C
G
T
A
G
C
T
A
C
G
T
T
C
G
A
T
C
G
A
T
C
G
A
C
T
G
A
C
T
A
G
A
C
G
T
T
C
A
G
C
G
A
T
T
C
A
G
  

|  |  |
| --- | --- |
| p-value: | 1e-55 |
| log p-value: | -1.278e+02 |
| Information Content per bp: | 1.714 |
| Number of Target Sequences with motif | 109.0 |
| Percentage of Target Sequences with motif | 3.40% |
| Number of Background Sequences with motif | 157.1 |
| Percentage of Background Sequences with motif | 0.36% |
| Average Position of motif in Targets | 100.2 +/- 34.7bp |
| Average Position of motif in Background | 102.8 +/- 74.5bp |
| Strand Bias (log2 ratio + to - strand density) | 0.4 |
| Multiplicity (# of sites on avg that occur together) | 1.00 |
| Motif File: | file (matrix) reverse opposite |

### Similar de novo motifs found

|  |  |  |  |  |  |  |  |
| --- | --- | --- | --- | --- | --- | --- | --- |
| Rank | Match Score | Redundant Motif | P-value | log P-value | % of Targets | % of Background | Motif file |
| 1 | 0.811 | A G T C C G T A A T G C G C A T A G C T A C G T C G A T G C T A C T G A T G C A T A G C G T C A G C T A G A T C | 1e-54 | -125.680015 | 3.21% | 0.32% | motif file (matrix) |
| 2 | 0.955 | G A T C C G T A A G T C C G T A A G T C C A G T A G C T A G C T G A C T T G C A C G T A G T C A | 1e-40 | -93.765639 | 1.90% | 0.11% | motif file (matrix) |
